# Comparative Transcriptome Analysis of Genes Involved in Sesquiterpene Alkaloid Biosynthesis in *Trichoderma longibrachiatum* MD33 and UN32

**DOI:** 10.3389/fmicb.2021.800125

**Published:** 2021-12-15

**Authors:** Xu Qian, Hui Jin, Zhuojun Chen, Qingqing Dai, Surendra Sarsaiya, Yitong Qin, Qi Jia, Leilei Jin, Jishuang Chen

**Affiliations:** ^1^College of Biotechnology and Pharmaceutical Engineering, Nanjing Tech University, Nanjing, China; ^2^Bioresource Institute for Healthy Utilization, Zunyi Medical University, Zunyi, China

**Keywords:** *Trichoderma longibrachiatum*, sesquiterpene alkaloids, mevalonate pathway, cytochrome P450, transcription factors

## Abstract

*Trichoderma longibrachiatum* MD33, a sesquiterpene alkaloid-producing endophyte isolated from *Dendrobium nobile*, shows potential medical and industrial applications. To understand the molecular mechanisms of sesquiterpene alkaloids production, a comparative transcriptome analysis was performed on strain MD33 and its positive mutant UN32, which was created using Ultraviolet (UV) mutagenesis and nitrogen ion (N^+^) implantation. The alkaloid production of UN32 was 2.62 times more than that of MD33. One thousand twenty-four differentially expressed genes (DEGs), including 519 up-regulated and 505 down-regulated genes, were identified. Gene Ontology (GO) and Kyoto Encyclopedia of Genes and Genomes (KEGG) pathway analysis revealed 139 GO terms and 87 biosynthesis pathways. Dendrobine, arguably the main sesquiterpene alkaloid the strain MD33 produced, might start synthesis through the mevalonate (MVA) pathway. Several MVA pathway enzyme-coding genes (hydroxy-methylglutaryl-CoA synthase, mevalonate kinase, and farnesyl diphosphate synthase) were found to be differentially expressed, suggesting that physical mutagenesis can disrupt genome integrity and gene expression. Some backbone post-modification enzymes and transcript factors were either discovered, suggesting the sesquiterpene alkaloid metabolism in *T. longibrachiatum* is a complex genetic network. Our findings help to shed light on the underlying molecular regulatory mechanism of sesquiterpene alkaloids production in *T. longibrachiatum*.

## Introduction

The plant *Dendrobium nobile* is well-known in Traditional Chinese Medicine (TCM) for containing a variety of active compounds, such as alkaloids, polysaccharides, phenols, terpenes, and flavonoids (Li et al., 2017). Such compounds have been proven to display a variety of biological activities, including moisturizing and cleansing the lungs, increasing saliva, and nourishing the stomach (Sarsaiya et al., 2019c,2020d; Lin et al., 2020). By quality criterion, dendrobine is among the most important sesquiterpene alkaloids of *D. nobile* (Kreis and Carreira, 2012). Extraction from plant is currently the main way to obtain dendrobine, which is not efficient and low yield. Therefore, the quest for a sustainable alternative source of high-value plant-metabolites is essential. This led to the discovery that endophytes can produce plant-derived compounds. The endophytes’ connection with their host plants is the consequence of extraordinary reworkings that enable the endophytes to grow in tandem with their plant associations. These endophyte groups are also responsible for the partial biosynthesis or broad distribution of secondary metabolites (SMs) produced by hosts (Sarsaiya et al., 2019b,2020c). Fungi may boost plant development, increase resistance to disease-causing pathogens, eliminate weeds, and improve plant tolerance to biotic and abiotic stressors. Additionally, they are very effective in producing large amounts of SMs (industrially significant bioactive natural chemicals) with pharmaceutical applications (Jain et al., 2019, 2021; Sarsaiya et al., 2019a,2020b). Frequently, pathway-specific regulatory proteins for fungoid SMs gene groups are discovered inside or next to the particular gene cluster. These proteins are unique in that they control the appearance of the whole gene cluster (Sarsaiya et al., 2019c,b; Qian et al., 2021). Our previous report was the first to identify *Trichoderma longibrachiatum* MD33, a dendrobine-producing endophyte isolated from *D. nobile* (Sarsaiya et al., 2020a).

The regulation of alkaloids biosynthetic pathway in *Dendrobium* plants has been the researchers’ main focus. Guo et al. (2013) identified several putative genes related to alkaloids biosynthetic pathway using transcriptomic analysis. In a study conducted by Li and associates (Li et al., 2017) on *D. nobile* stems infected with the mycorrhizal fungus MF23, the results of large-scale transcriptome sequence revealed that dendrobine-related genes were categorized into two clusters for dendrobine skeleton biosynthesis and modification. Multiple transcription factors (TFs), including the NAC, MYB, and bHLH families were also found to be up-regulated when MeJA was used (Chen et al., 2019). Although some dendrobine-related genes have been identified through transcriptome and bioinformatic analysis, it is still difficult to propose a complete biosynthesis pathway based on the candidate genes due to the complexity of plant genome, sophisticated genetic manipulation, and prohibitive cost. Endophytes are considered as potential alternatives for terpenoid bioproduction due to their long-term harmony and coevolution with plants (Venugopalan and Srivastava, 2015). However, the main obstacles to the commercialization of endophytes are the low inner catalytic activity of plant-derived enzymes, lack of gene information regarding the biosynthesis pathway, and the silence of SM gene clusters (Gupta et al., 2020; Qiao et al., 2020; Chen et al., 2021). Modern RNA-sequencing methods enable researchers to gain a quick and comprehensive understanding of the plant-derived compound biosynthesis pathway in endophytes, and determine the association between each individual gene and a phenotype (Zhang et al., 2015; Qiao et al., 2020).

To fully comprehend the sesquiterpene alkaloids synthesis pathway in endophytic fungi, a stable and positive mutant strain UN32 obtained from the strain MD33 was studied. In total, more than 60 genes involved in sesquiterpene alkaloids synthesis and regulation were discovered through comparative transcriptome analysis between the strain MD33 and UN32. Our findings elucidate the possible mechanism that controls the accumulation of sesquiterpene alkaloids in the endophyte *T. longibrachiatum*.

## Materials and Methods

### Strain and Culture Conditions

The dendrobine-producing strain *T. longibrachiatum* MD33 was isolated from *D. nobile* (Sarsaiya et al., 2020a), and kept in potato dextrose agar medium (g/l): potato: 200; dextrose 20 and agar 20. The potato dextrose broth (PDB) means PDA medium without agar, and is used for co-culturing mutants with *D. nobile* protocorm. The protocorm was induced from wild *D. nobile* capsule picked from Chishui City and kept in proliferation medium (Qian et al., 2021). After culturing for 30 days, the aseptic and healthy protocorm were selected to co-culture.

### Extraction of Total Alkaloids

The total alkaloids (TAs) were extracted using Chen’s previously reported technique with minor modifications (Chen et al., 2019). After 14 days of incubation at 28°C, the mycelia of wild type and mutants were isolated from the PDB medium and dried at 45°C for 48 h. All dried powder samples (0.50 g) were extracted with hydrochloric acid solution (25 ml, 2%, v/v) for 10 min in an ultrasonic cell-crushing device at room temperature, and then steeped for 12 h before being adjusted to a pH of 10. An equivalent amount of dichloro-methane was added for extraction, and the bottom layer was collected and dried. After resolving the dried residue in 5 ml of dichloromethane, the TA content was measured using the potassium biphthalate buffer (pH 4.5) and bromocresol green (Jiao et al., 2018).

### Mutagenesis Assay of Total Alkaloids-Producing Strain

**FIGURE 1:**
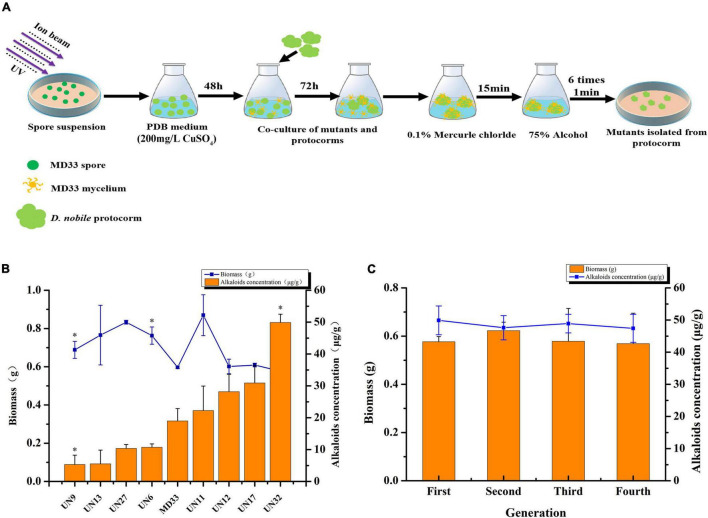
Ultraviolet rays and N^+^ implantation of *T. longibrachiatum* MD33 to increase total alkaloids production. **(A)** Schematic illustration of the mutants screening process. **(B)** Comparison of total alkaloids production and biomass between MD33 with its mutants. **(C)** Dry weight (g) and total alkaloids production (μg/g) of UN32 strain grown for four successive generation. Asterisks indicate significant differences; one asterisk represents *p* < 0.05.

To produce positive mutant with enhanced TA production, Ultraviolet (UV) irradiation and N^+^ implantation mutagenesis were employed (Figure 1A). To begin, the MD33 spore on PDA was transferred into 10 ml PDB with 0.1% tween-80 and the cell concentration was adjusted to 10^6^ spores/ml. This suspension was kept at room temperature for 12 h followed by UV irradiation (15 cm apart from the UV source) for 2 min, which caused 99% killing of spores. Then the treated suspension (100 μl) was pipetted and uniformly covered onto a sterile empty petri dish. After air drying, N^+^ ion beam implanter (LZD-900, China) was used to mutate the strain. The parameters were set at nitrogen ion density of 15 × 10^14^cm^2^, beam energy of 30 keV, current of 400 μA, and vacuum degree of 10^–3^ Pa in the target chamber. The control sample was also placed in the irradiation chamber but not irradiated. To maintain screening pressure and avoid contamination, the irradiated spore suspension was put to PDB medium containing 200 mg/L CuSO_4_ (minimal bactericidal concentration) for 48 h. The *D. nobile* protocorms were introduced to the flask and co-cultured for 72 h with the mutants. Following that, the mutants were isolated from the protocorms using the technique described previously (Sarsaiya et al., 2020a), and the positive mutant strain was designated as UN32.

### RNA Extraction

Each strain’s total RNA was extracted using the RNA extraction kit (TSINGKE and TSP401). Following that, DNAase was used to further break down the genomic DNA contamination. Gel electrophoresis and Qubit2.0 were used to determine the integrity and concentration of isolated RNA, respectively.

### Library Sequencing, Assembly, and Analysis

The preparation of the transcriptome library was conducted by KEGENE Company (Shandong, China), and sequenced on the HiSeq 4000 Illumina platform (Illumina, United States). The unique transcripts were obtained after removing the low number of reads from the raw data. We annotated all unigenes against a full set of BLAST searches to find the most descriptive annotations, including the NR (*e*-value = 1e-5), NT (*e*-value = 1e-5), KOG (*e*-value = 1e-3), PFAM (*e*-value = 0.01), Swiss-Prot (*e*-value = 1e-5), ITAK (TF prediction software based on hmmscan) (Soni et al., 2021), Gene Ontology (GO) (*e*-value = 1e-6), and Kyoto Encyclopedia of Genes and Genomes (KEGG) (*e*-value = 1e-10) database. To calculate and normalize the transcript abundance of the unigenes in each sample, the FPKM method was adopted (Li and Dewey, 2011). Following that, a false discovery rate (FDR) calculation was conducted by the edgeR package (Robinson et al., 2010). Differentially expressed genes (DEGs) were defined as FDR < 0.05 and absolute value of fold change > 2.

### qRT-PCR Validation

qRT-PCR was performed to validate the accuracy of RNA-seq data. All selected genes were compared with 2^–ΔΔCt^ values (Zhao et al., 2021). Total RNA was extracted by TsingZol Total RNA Extraction Reagent (TSINGKE and TSP401), and evaluated for integrity. The cDNA synthesis kit (TSINGKE and TSK302M) was used to synthesize cDNA from 1 μg of RNA. This kit can remove the genomic DNA. The gene primers used are listed in Supplementary Table 5 with GAPDH served as a reference gene. For gene expression analysis, the Applied Biosystems StepOnePlus™ Real-Time PCR System was used. qRT-PCR amplification was performed in 20 μL reactions containing 10 μL 2 × TSINKE^®^ Master qPCR Mix (SYBR GREEN), 1 μL cDNA, and 0.4 μM of each primer. The qRT-PCR reaction procedure was performed as follows: 95°C for 1 min; 40 cycles of 95°C for 10 s, and 60°C for 30 s. A melting curve analysis was carried out by gradually increasing the temperature from 60 to 95°C.

### Statistical Analysis

The data acquired was processed and analyzed *via* student’s *t*-test using the statistical tool SPSS 20.0. At least three biological replicates were performed for each analysis and the results and errors are the mean and SD, respectively, from three replicates. *P*-value < 0.05 was considered as statistical significant.

## Results

### Determination of Total Alkaloids in MD33 and UN32

In this study, UV radiation and N^+^ implantation were employed to induce the genetic mutation of the strain MD33. The positive mutant strain UN32 was chosen based on its TA content and biomass. On day 14, the biomass of UN32 is close to that of MD33. However, the TAs content rose substantially about 2.63 times, from 18.97 to 49.91 μg/g (Figure 1B). After four consecutive generations, the biomass and TAs content of positive strain UN32 was stable (Figure 1C).

### Sequencing, Assembly, and Annotation

The transcriptomes of the original strain *T. longibrachiatum* MD33 and its mutant *T. longibrachiatum* UN32 were sequenced with Illumina sequencing technology using three independent samples. After quality control of raw reads, the clean reads were obtained (Supplementary Table 1). Clean reads were assembled into 6,922 genes (mean length = 4476 bp) and 33,030 transcripts (mean length = 6110 bp) (Supplementary Tables 2, 3). The size distributions of transcripts and genes were analyzed. Out of 33,030 transcripts, 294 transcripts (0.89%) were 200–500 bp in length, 4,801 transcripts (14.53%) were 500–2000 bp long, and 27,935 transcripts (84.57%) were longer than 2,000 bp. Genes longer than 2,000 bp occupied the largest proportion of all the assembled genes (Supplementary Table 2).

When compared with the NR database by BLASTX search, 77.33% of unigenes were found to be annotated. In addition, 1,940 (28.03%) unigenes were annotated into GO terms, while 3,906 (56.43%) unigenes were annotated with KEGG. 1,971 (28.47%), 5,106 (73.76%), and 5,244 (75.47%) unigenes were annotated in the Swiss-Prot, PFAM, and KOG databases, respectively. In total, 5,494 unigenes (79.37%) were annotated in at least one database and 1,499 unigenes (21.66%) were annotated in all databases, indicating that the annotation was of relatively good quality (Supplementary Table 4). These annotations provided a valuable resource for further research on specific processes, functions, and pathways in *T. longibrachiatum* MD33.

#### Kyoto Encyclopedia of Genes and Genomes and Gene Ontology Classification of Differentially Expressed Genes

**FIGURE 2:**
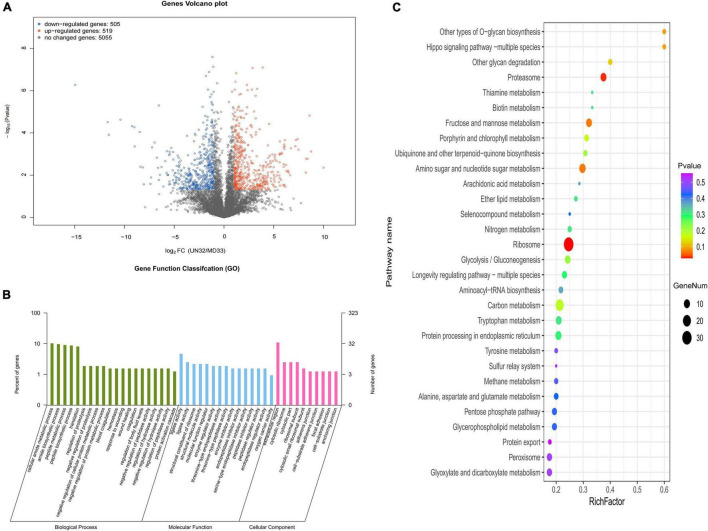
Transcriptional variation between strain MD33 and UN32. **(A)** Significance analysis of all DEGs between the strain MD33 and UN32 by a volcano plot. **(B)** GO enrichment analysis. **(C)** KEGG enrichment analysis.

The final read density for each gene was normalized to screen the DEGs between MD33 and UN32. The result was shown as a volcano plot in Figure 2A. Altogether, 1,024 DEGs (519 up-regulated and 505 down-regulated) were identified. To learn more about the functional characterization of DEGs, homology searches were used to classify the obtained putative genes into biological processes, cellular components, and molecular functions (Figure 2B). The most enriched GO terms were the “nitrogen compound metabolic process” (685 genes), “macromolecule metabolic process” (480 genes), “organonitrogen compound metabolic process” (468 genes), “protein-containing complex” (287 genes), “ribonucleoprotein complex” (173 genes), “ribosome” (128 genes), “ligase activity” (51 genes), “endopeptidase activity” (29 genes), and “catalytic activity, acting on a tRNA” (26 genes). Furthermore, KEGG analysis assigned 2,320 genes to 87 pathways, the “Proteasome” (ko03050) and “Ribosome” (ko03010) pathway being the most significantly enriched (Figure 2C).

#### Expression Changes of Genes in Sesquiterpenoid Metabolism Pathway

**FIGURE 3:**
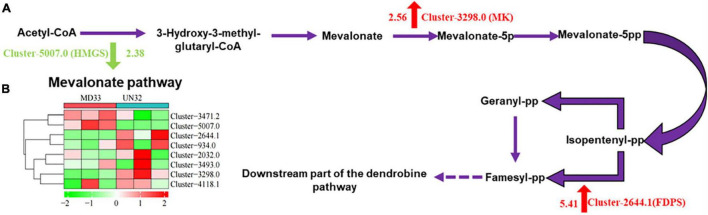
Heat map of transcript abundance for genes related to MVA pathway. **(A)** Schematic illustration of MVA biosynthetic pathways in MD33. Abbreviations, as quoted in the figure, are as follows. HMGS, hydroxymethylglutaryl-CoA synthase; MK, mevalonate kinase; FDPS, farnesyl diphosphate synthase. **(B)** Expression changes of the genes associated with MVA pathway in the strain MD33 and UN32. Red indicates up-regulated genes and green indicates down-regulated genes.

The mevalonate (MVA) pathway is well established in fungi, and several reports of dendrobine suggest its sesquiterpene origin. In our study, three DEGs were mapped into the MVA pathway, including 3-hydroxy-3-methylglutaryl-CoA synthase (HMGS), mevalonate kinase (MK), and farnesyl diphosphate synthase (FDPS) (Figure 3A). Gene expression levels were represented as a heat map based on FPKM values (Figure 3B). It was found that *hmgs* (Cluster-5007.0) decreased expression (log2 fold change = 2.38) in strain UN32. However, the gene *mk* and *fdps* boosted their expression (log2 fold change = 2.56 and 5.41, respectively). In particular, the gene *fdps* was found only expressed in the strain UN32 based on FPKM value, suggesting that physical mutagenesis might disrupt gene expression.

#### Comparison of the Expression Levels of Post-modification Enzymes

**FIGURE 4:**
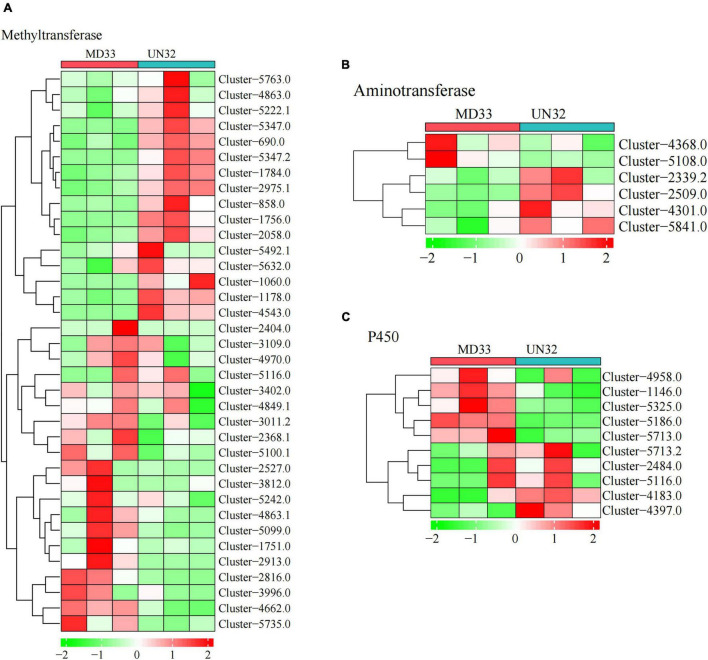
Heat map analysis of genes in backbone post-modification. **(A)** Heat map analysis of methyltransferases. **(B)** Heat map analysis of aminotransferases. **(C)** Heat map analysis of Cytochrome P450 family.

The post-modification enzymes involved in the dendrobine biosynthesis pathway mainly included cytochrome P450, methyltransferase and aminotransferase. By analyzing the DEGs, We obtained 10 putative P450 unigenes (Figure 4C), of which four unigenes (Cluster-1146.0, Cluster-4183.0, Cluster-5186.0, and Cluster-5325.0) expressed differently between MD33 with UN32 (Table 1). The Cluster-4183.0 was the only up-regulated gene (log2 fold change = 3.34) among them. Aminotransferase and methyltransferase as another two important enzymes involved in post-modification were also analyzed. Clustering analysis revealed that only one aminotransferase (Cluster-2509.0) increased expression (log2 fold change = 1.98-fold) in strain UN32 (Figure 4B and Supplementary Table 6). Overall, 16 putative methyltransferases were identified, with half of them showing enhanced expression in the UN32 strain (Supplementary Table 7). Among the genes with increased expression, three (Cluster-1060.0, Cluster-4543.0, and Cluster-5347.2) like the *fdps* gene were solely expressed in the strain UN32 (Figure 4A).

**TABLE 1:** Cytochrome P450 families identified from the DEGs between the strain MD33 and UN32.

Gene ID	MD33-FPKM			UN32-FPKM			Log2FC	Nelson’s P450 name	CYP450 clans	Identity	*e*-value
	1	2	3	1	2	3					
Cluster-1146.0	38.27	49.09	39.85	29.25	16.22	13.19	−1.131	CYP5077A1	CYP531	46.56%	1e-125
Cluster-4183.0	0	0	10.13	14.15	15.77	11.71	3.339	CYP62A1	CYP62	70.59%	0
Cluster-5186.0	221.99	198.09	199.82	38.68	52.64	54.77	−2.2066	CYP53C2	CYP53	71.27%	0
Cluster-5325.0	30.46	61.62	45	15.98	13.54	7.02	−1.831	CYP570D1	CYP507	56.12%	1e-156

#### Differentially Expressed Genes Related to Transcription Factors

Transcription factors play a crucial role in the SM biosynthesis pathway in fungi, and several of them have been reported. In total, 46 putative TF-encoding genes belonging to 11 major TF families were analyzed (Table 2). The Zn-Clus (20 genes) and the zinc finger protein contained the most members, including 2 C2C2-GATA genes, 6 C2H2 genes, and 3 C3H genes. Among these TF genes, 7 Zn-Clus genes, 5 C2H2 genes, and 2 SET genes were found to be up-regulated in the UN32 strain.

**TABLE 2:** Transcription factors identified from the DEGs between the strain MD33 and UN32.

TFs	Number of transcripts	Up-regulated	Down-regulated	TFs	Number of transcripts	Up-regulated	Down-regulated
bHLH	1	0	1	HB-other	2	1	1
C2C2-GATA	2	1	1	HSF	1	0	1
C2H2	6	5	1	Jumonji	2	0	2
C3H	3	1	2	NF-YC	1	0	1
Coactivator	1	1	0	SET	3	2	1
GNAT	3	1	2	SNF2	1	1	0
Zn-Clus	20	7	13				

#### qRT-PCR Validation of RNA-seq Results

**FIGURE 5:**
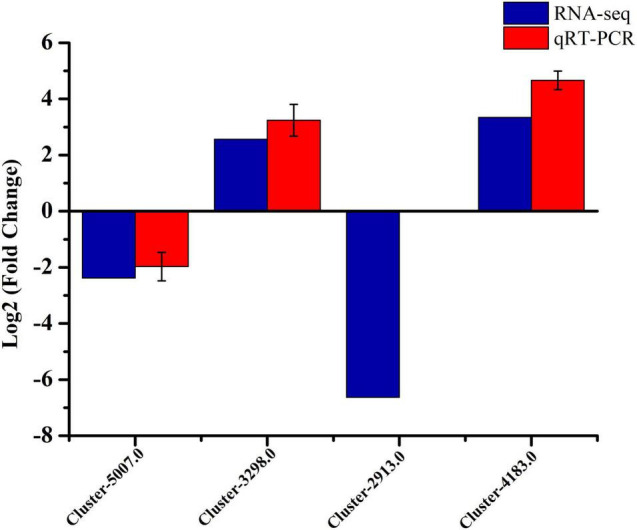
qRT-PCR verification of the RNA-seq data of four selected DEGs in the strain MD33 and UN32. The blue columns represent RNA-seq data and the red columns represent qRT-PCR validation result. Values of qRT-PCR validation are presented as the log2 (fold change) ± SE.

The qRT-PCR was conducted for validation, and four DEGs were randomly selected from RNA-seq data. These genes included *hmgs* (Cluster-5070.0), *mk* (Cluster-3298.0), *methyl* (Cluster-2913.0), and *cyp450* (Cluster-4183.0). The results showed that the qRT-PCR was basically consistent with the RNA-seq results, apart from Cluster-2913.0 (Figure 5). The measured qRT-PCR expression of Cluster-2913.0 gene was similarly low in the strain MD33. Based on transcriptomic data, Cluster-2913.0 (methyltransferase) was only missing in the strain UN32, and this could be attributed to the quality of the libraries or their sequencing. In general, the RNA-seq data is trustworthy.

## Discussion

*Dendrobium nobile* is a medical and edible plant of great economic value, exhibiting many physiological functions (He et al., 2020). Chen and associates (Chen and Chen, 1935) were the first to identify and report alkaloids in *Dendrobium*. Modern research has demonstrated that alkaloids such as dendrobine possess therapeutic and pharmacological properties. As a result, scientists have paid attention to learning their biogenetic pathways and pharmaceutical mechanisms (Li et al., 2017).

Transcriptome sequencing is an effective tool to investigate the synthesis and metabolic pathways of specialized metabolites. Recently, a hypothesized sesquiterpene alkaloid biosynthetic pathway was proposed. It comprises three steps: formation of the precursor (isopentenyl diphosphate), ring closure for the sesquiterpene skeleton, and modification and oxidation. Chen et al. (2019) reported that the MVA and methyl-D-erythritol 4-phosphate (MEP) pathway, upstream of the *Dendrobium* alkaloids biosynthetic pathway, were probably the main sources of IPP. The acetyl-CoA acetyltransferase, phosphomevalonate kinase, and diphosphomevalonate decarboxylase in the MVA pathway might be positively linked with dendrobine accumulation in *D. nobile* (Li et al., 2017). In this study, only MK and FDPS increased expression in the mutant strain. The hydroxymethylglutaryl-CoA synthase, another important synthesis enzyme in *Dendrobium officinale*, was found to have decreased expression in UN32. We hypothesized that the increased alkaloid content might be related to the increased expression of the *fdps* gene, because the *fdps* gene catalyzed the formation of farnesyl diphosphate from IPP. Similarly, in the study conducted by Li et al. (2017), the TPS21 enzyme facilitated the formation of the skeleton of murolene-type sesquiterpene from farnesyl diphosphate.

Chemical modification catalyzed by enzymes is responsible for structural diversity in alkaloids, such as CYP450-mediated oxidation and hydroxylation reactions. Cytochrome P450 (CYP450) enzymes, belonging to a superfamily of monooxygenase, have been identified in *Dendrobium* genus (Mou et al., 2021). Working on MeJA-induced alkaloids accumulation in *D. officinale*, Chen and associates (Chen et al., 2019) identified 59 CYP450s involved in alkaloids biosynthesis through phylogenetic tree and gene expression pattern analysis. Yuan (Yuan et al., 2018) also discovered that some CYP450s, including CYP71, CYP3A, and CYP4 family members, are associated with alkaloid hydroxylation steps in *Dendrobium huoshanense*. 4 putative genes (Cluster-1146.0, Cluster-4183.0, Cluster-5186.0, and Cluster-5325.0) from 4 clans (CYP531, CYP62, CYP53, and CYP507 clan) were identified in our RNA-seq data. This result slightly differs from Chadha’s report (Chadha et al., 2018), which states that a total of 477 CYP450s were identified and annotated in seven *Trichoderma* species, and their evolutionary relationships were analyzed. In the *T. longibrachiatum* ATCC18648 genome, 55 CYP450s were found and annotated. The CYP53 clan, in particular, was found in all *Trichoderma* species except *T. longibrachiatum*, and the CYP62 clan was the other CYP450 protein only found in *Trichoderma harzianum* (Chadha et al., 2018). The CYP53 and CYP62 clan were discovered for the first time in *T. longibrachiatum* in our study. The DEG Cluster-5186.0 and Cluster-5325.0 belong to the CYP53 and CYP62 clans, with 71.27 and 70.59% identity, respectively. Our findings could point to a novel role of CYP53 and CYP62 clans in accumulation of alkaloids. According to Li et al. (2017), aminotransferases and methyltransferases are the other two modification enzymes required to complete the chemical structure of alkaloids, particularly in dendrobine. In this study, several DEGs were annotated as aminotransferases and methyltransferases. Although the annotation for these genes indicates roles in the alkaloid biosynthetic pathway in *T. longibrachiatum* MD33, we have not been able to pinpoint the precise role of the protein encoded by those genes.

Substantial evidence has proved that TFs are also involved (Goklany et al., 2013; Yamada and Sato, 2013). The FTFD database records five different TF classes, including the fungus-specific Zn(II)2Cys6 class, the C2H2 zinc finger class, the Bzip class, the bHLH class, and the GATA-type classes. These TFs have been discovered in *Trichoderma atroviride*, *Trichoderma virens*, and *Trichoderma reesei*, and have functions in controlling cellular development, sugar and amino acid metabolism, nutrient utilization, chromatin remodeling, and various stress responses (MacPherson et al., 2006; Tian et al., 2011; Yin and Keller, 2011). In the present study, some TFs related to alkaloids biosynthesis in *T. longibrachiatum* were first identified. The Zn-Cluster TFs with the highest number of unigenes were significantly up-regulated in the positive strain UN32. Apart from the Zn-Cluster TFs, C2H2 (6 genes), C3H (3 genes), C2C2-GATA (2 genes), and bHLH (1 gene) TFs also changed expression under the strain UN32, suggesting their potential roles in alkaloid biosynthesis. Four DEGs were selected randomly for validation by qRT-PCR methods. All genes were essentially consistent between the sequencing data with qRT-PCR trials (Figure 5).

## Conclusion

We obtained an alkaloid-producing endophytic fungus, UN32, by physical mutagenesis, with a relatively higher TA yield, conducted transcriptome analysis between two TA-producing fungi with different TA production and identified the potential mechanism for the changes in production. The results showed that there were 1,024 DEGs, many of which were associated with the postulated alkaloid biosynthesis pathway in *T. longibrachiatum* MD33. Several genes involved in the MVA pathway were significantly up-regulated, suggesting an active supply of precursors for alkaloid production. Additionally, numerous CYP450s, aminotransferases, methyltransferases, and TFs were discovered, giving various possibilities for elucidating the MD33 alkaloid biosynthesis route. Our results advance our knowledge of the processes governing the accumulation of alkaloids in endophytic fungus.

## Data Availability Statement

The datasets presented in this study can be found in online repositories. The names of the repository/repositories and accession number(s) can be found below: https://www.ncbi.nlm.nih.gov/, PRJNA763081.

## Author Contributions

XQ, QD, ZC, HJ, and YQ: investigation. JC: project administration. QJ: resources. LJ: supervision. XQ: writing – original draft. SS and JC: writing – review and editing. All authors contributed to the article and approved the submitted version.

## Conflict of Interest

This study received funding from Guizhou Science and Technology Corporation. The funder had involvement with the study design. The authors declare that the research was conducted in the absence of any commercial or financial relationships that could be construed as a potential conflict of interest.

## Publisher’s Note

All claims expressed in this article are solely those of the authors and do not necessarily represent those of their affiliated organizations, or those of the publisher, the editors and the reviewers. Any product that may be evaluated in this article, or claim that may be made by its manufacturer, is not guaranteed or endorsed by the publisher.
